# Accelerating care, capacity and equity in automated insulin delivery systems for New Zealanders with type 1 diabetes: the ACCESS-AID study protocol

**DOI:** 10.1007/s40200-026-01864-0

**Published:** 2026-03-02

**Authors:** Jennifer T. Gale, Alisa Boucsein, Jonathan Williman, Claire Lever, Hamish Crocket, Ofa Dewes, Shelley Rose, Helen Snell, Chunhuan Lao, Martin de Bock, Craig Jefferies, Rosemary Hall, Shekar Sehgal, Shirley Jones, Ryan Paul, Benjamin J. Wheeler

**Affiliations:** 1 Department of Paediatrics and Child Health, Dunedin School of Medicine, University of Otago, Dunedin, New Zealand; 2 School of Sport, Exercise and Health, Auckland University of Technology, Auckland, New Zealand; 3 Biostatistics and Computation Biology Unit, University of Otago, Christchurch, New Zealand; 4 Aotearoa Diabetes Collective, Waikato, New Zealand; 5 School of Health Equity and Innovation, Division of Health, University of Waikato, Hamilton, New Zealand; 6 Centre of Methods & Policy Application in the Social Sciences, University of Auckland, Auckland, New Zealand; 7 The Cause Collective, Auckland, New Zealand; 8 Diabetes & Endocrinology Service, Health NZ Te Whatu Ora MidCentral, Palmerston North, New Zealand; 9 Department of Paediatrics, University of Otago, Christchurch, New Zealand; 10 Department of Paediatrics, Health NZ Te Whatu Ora, Waitaha, Christchurch, New Zealand; 11 Starship Child Health, Te Whatu Ora Te Toka Tumai, Auckland, New Zealand; 12 Liggins Institute, University of Auckland, Auckland, New Zealand; 13 Health NZ Te Whatu Ora Capital Coast and Hutt Valley and Tairāwhiti, Wellington, New Zealand; 14 Department of Medicine, University of Otago Wellington, Wellington, New Zealand; 15 Health NZ Te Whatu Ora Waitematā, North Shore Hospital, Auckland, New Zealand; 16 School of Medicine, University of Auckland, Waitematā Clinical Campus, Auckland, New Zealand; 17 Waikato Regional Diabetes Service, Health NZ Te Whatu Ora, Waikato, Hamilton, New Zealand; 18 Health NZ Te Whatu Ora Southern, Dunedin, New Zealand

**Keywords:** Health delivery, Public health, Type 1 diabetes, Health inequities, continuous glucose monitoring

## Abstract

**Purpose:**

Automated insulin delivery (AID) systems are the gold standard for managing type 1 diabetes (T1D), yet access remains inequitable due to funding disparities, workforce limitations, bias, and geographic barriers. The ACCESS-AID study aims to implement a new model of care by using a remote ‘Hub’ to deliver prioritised training and support to those most in need and to improve workforce capacity by working in partnership with New Zealand’s National Public Health service.

**Methods:**

Eligible participants include all individuals with T1D and eligible people with pancreatogenic/Type 3c diabetes). Enrolment will use a prioritisation score. After informed consent and baseline assessments, participants receive one-day AID training (in-person or remote) by certified, industry provided trainers, followed by 12-weeks of structured support from Hub staff. The primary outcome is implementation effectiveness. Secondary outcomes: clinical and psychosocial impacts, safety, nutrition education effectiveness, and qualitative insights. CGM metrics and HbA1c will be assessed at baseline and 12-weeks, and CGM again at 24-weeks. Hub staff will receive training in AID management, complete self-efficacy assessments, and participate in interviews.

**Conclusion:**

This model offers a novel, scalable and equity-focused approach to diabetes technology care, which will enhance outcomes for people with diabetes and inform future service delivery for other long-term conditions.

## Background

Automated insulin delivery (AID) systems are globally recognised as the gold standard for managing glucose levels in people with type 1 diabetes (T1D) [1, 2]. These systems link an insulin pump, continuous glucose monitor (CGM) and computer algorithm to semi-automate insulin delivery. AID systems have been shown to greatly improve glycaemic outcomes [3]. For example, in a randomised controlled trial of children and youth aged 7 to 25 years with above-target glycemia, HbA1c decreased by 2.5% (95% CI −3.1 to 1.8 [−27mmol/mol, 95% CI −34 to −20] *p* < 0.001) and time in range (TIR) increased by 35% (95% CI 28 to 41%) after using AID for 13-weeks, compared to the control group (multiple daily injections or continuous subcutaneous insulin infusion) [4].

Despite this evidence, in Aotearoa New Zealand (herein referred to as NZ) and worldwide, access to these advanced diabetes technologies is slow and inequitable [5–7]. Until recently in NZ, the main barriers to access included the lack of publicly funded CGM and restrictive eligibility criteria for insulin pump funding and consumables particularly for adults, people from low socioeconomic backgrounds, or ethnic minorities [5] with similar trends observed worldwide [8]. However, in October 2024, funding in New Zealand was expanded to include CGM and pumps for all people with type 1 and related forms of diabetes, enabling broader use of AID systems. Despite advances in technology and liberalisation of funding, many barriers to access remain. Historical treatment guidelines prioritised those who had more in-target glycemia and diabetes management skills [9, 10], effectively gatekeeping this technology from individuals who experience the greatest absolute benefits. Furthermore, workforce constraints, and sometimes overly complicated training and support pathways remain key barriers [11, 12] with evidence from the UK highlighting funded practitioner time as a key barrier for supporting individuals to use this technology [13]. Now more than ever, there is an urgent need to provide efficient and equitable access to AID for the thousands of individuals estimated to seek this technology in the coming years.

Although global evidence supports the use of telehealth for effective management of both diabetes [14] and other chronic disease [15, 16], particularly to improve patient access [17, 18], uptake of telehealth models has been slow. Diabetes nursing telehealth services are just as effective as standard care models with evidence for sustained glycaemic improvements [19] demonstrating the potential for this model of care. However, current telehealth models have been limited to ongoing management rather than initiation of new technology. The Accelerating Care, Capacity and Equity in Automated Insulin Delivery Systems for New Zealanders with Type 1 Diabetes (ACCESS-AID) Study, aims to simplify AID training by utilising a remote health care team to prepare and support individuals regardless of their physical location, to begin AID following structured online patient education, and a highly effective in-person, single day training session provided by certified pump trainers. This approach aims to efficiently and safely speed up training and access to AID, and has been used previously by our research team to successfully train high-risk and priority populations across the lifespan onto AID systems [20–23]. Furthermore, evidence from our research group indicates that access to modern diabetes technologies is an effective equaliser of ethnicity-based disparities in diabetes outcomes, and thereby is an effective equity tool [6, 24]. Therefore, the ACCESS-AID study will use a prioritisation score to ensure equitable access to overcome some of the documented barriers in the current health care system.

This study will also include a ‘train-the-trainer’ model, which is an effective method for knowledge and clinical practice dissemination [25, 26], whilst also being cost-effective and able to be moulded to local requirements [27]. Clinical staff, such as registered nurses, physicians, pharmacists and dietitians from existing diabetes related health services will be seconded to the ACCESS-AID Hub, during which time they will be provided with training and support to gain confidence and skills related to AID management. This is important as a lack of knowledge and confidence in T1D advanced technologies has been identified as a barrier to access [28, 29]. Therefore, while the aims are to improve efficiency and equity of access to technology, this study will also mentor and train staff to increase capacity throughout the public health system. Furthermore, as previous success in AID has undoubtedly been supported by the fact that the recipients of AID are within a research project receiving close support and attention [23, 30], it is important to evaluate whether we can safely integrate these training models into standard clinical care to support existing teams and training methods.

This study builds on our wider research groups’ portfolio of experimental studies and moves this work into the implementation phase to bring this new model of care into mainstream clinical practice. This study is being conducted in partnership with Health New Zealand/Te Whatu Ora, the national public health care provider, and aims to deploy a novel national remote “hub” model (herein referred to as the Hub), to prioritise access to AID for those with greatest need. This new model of care is designed to specifically address the well documented barriers to access [29, 31–33], with a specific focus on priority populations living with T1D such as those living rurally, Māori and Pacific peoples, those with less in-target glycemia and/or more diabetes-related complications. Furthermore, we anticipate that this model of care may provide a framework for the management of other areas of diabetes care, or other chronic conditions, both nationally and worldwide.

### Objectives

This study has four main objectives: (1) To determine the effectiveness and safety of a remote Hub to start AID and address inequities in care; (2) To investigate using qualitative methodologies whether the Hub is acceptable for both people and whānau (families) living with T1D and their health care teams, and how the model can be improved; (3) To determine whether inviting diabetes care providers to partake in the Hub is effective in improving knowledge about, and training methods for AID; (4) To determine the cost effectiveness of the Hub in starting AID compared to current models across districts in NZ.

## Methods

### Trial conduct

#### Study design

**Fig. 1:**
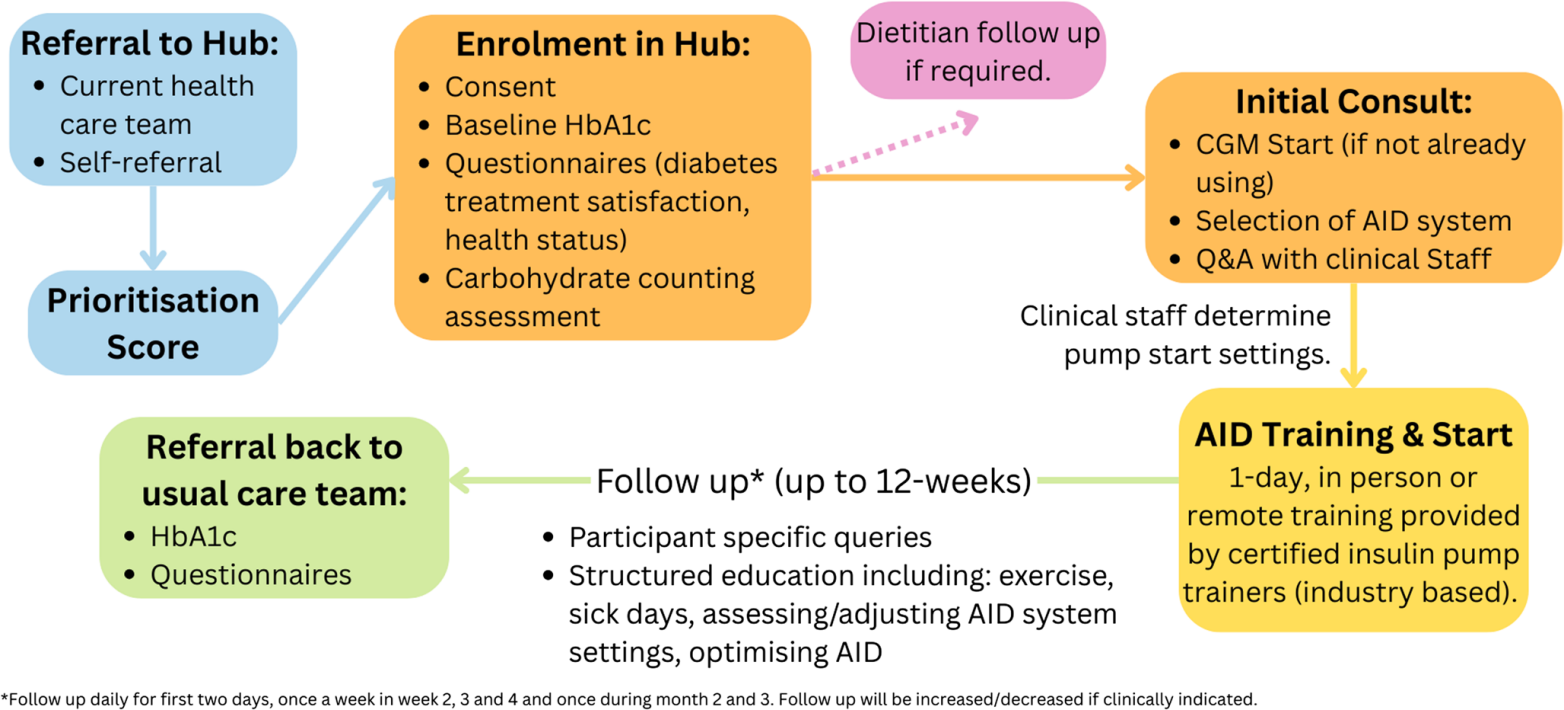
ACCESS-AID study design summary

This is a quasi-experimental, single-arm study which will assess a new model of AID health care delivery in NZ. The new model of health care will be the remote Hub which will be deployed to provide prioritised national AID set up and support, before transferring participants back to their usual care teams after a maximum of 12 weeks (See Fig. 1 for summary of study design). The duration from referral to enrolment may vary depending on participants prioritisation score (outlined below in Sect. 2.3.2).

#### Design of remote hub

The Hub will be staffed by a multidisciplinary clinical team from various regions across NZ. Clinical staff members will be registered nurses/diabetes nurse specialists ideally with prescribing authorisation. Endocrinologists and other allied health professionals involved in the care of patients with T1D (e.g., prescribing pharmacists and dietitians) will also be part of the team. Most direct participant contact will be conducted by the nursing and allied health staff (as compared to endocrinologists). Those working in primary care may also be employed to staff the Hub. Appropriate supervision will be provided.

#### Consent

Participants will be sent an email/text to complete online informed consent using REDCap (REDCap software, Research Electronic Data Capture, production server version 14.3.0, Vanderbilt University, Nashville, TN, USA). The email, and online portal will have a copy of the participant information sheet. If unable to be completed online, a copy of the participant information sheet and consent forms will be sent via post to the participant to complete. Participants will be encouraged to discuss participation with their whānau (family) and usual care providers, and to contact the research team with any questions prior to providing informed consent.

### Study population

All people with T1D, and people with pancreatogenic or Type 3c diabetes who meet the public funding access criteria for an insulin pump and CGM [34], will be eligible to participate. Speed of access will be based on their prioritisation score. Furthermore, interested people must have a suitable health care team (determined by study clinical staff) who are available and willing to provide ongoing care to the person with diabetes once their participation in the study is complete. Existing healthcare teams will be notified when their patient has been enrolled and updated throughout their time enrolled in the Hub. Individuals who are pregnant will not be eligible to participate as these individuals will be prioritised through existing health delivery pathways. Additionally, those who have non-diabetes related, active moderate-to-severe health and/or mental health issues, will be referred to appropriate health care providers first for support and management, before transitioning to diabetes technology (at the discretion of the clinical team). In situations where additional requirements are identified, eligibility will be assessed on a case-by-case basis to ensure safety and support of transitioning to AID (for example, severely impaired vision or recent acute medical event).

#### Recruitment

Interested individuals may be referred from any primary or secondary care provider in NZ (through word of mouth and dissemination within the wider research teams clinical networks), or interested individuals can approach the study team directly. The referral form will include a range of required information including demographics, recent anthropometric measurements, diabetes history and relevant clinical history. Regardless of method of referral, the study team will confirm agreement from participants’ usual diabetes health care provider for ongoing care after AID training and discharge from the study team.

### Procedures

#### Intervention

All participants enrolled in the study will receive the intervention as this is a single-arm study. Once enrolled, participants will be supported to start an AID system and be followed up over a period of 12 weeks (see study schedule in Table 1).

**Table 1:**
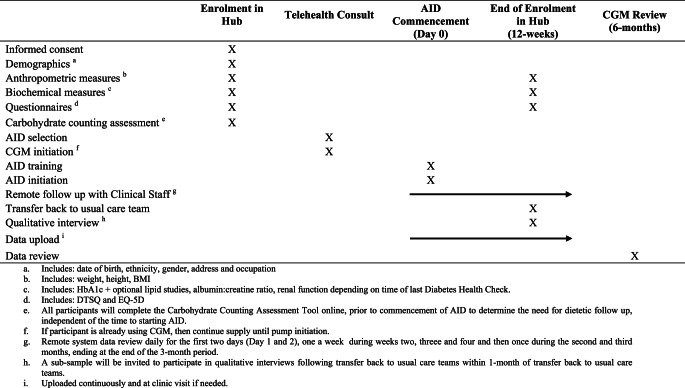
ACCESS-AID study schedule

#### Prioritisation score

Data collected from the referral form will be used to calculate a prioritisation score (Table 2). Given the substantial waiting lists for AID access across NZ, the prioritisation score is designed to ensure current inequities in access to AID are addressed and those who may benefit the most are prioritised.

**Table 2:** Study prioritisation framework

Priority items:	Potential Score
Māori and/or Pacific ethnicity	2 points
HbA1c > 86 mmol/mol (10%) ^1^	2 points
Living rurally ^2^	1 point
HbA1c 58–85 mmol/mol (7.5 to 10%)	1 point
Severe hypoglycaemia in last 2 years	1 point
Age < 25 years ^3^	1 point
Known diabetes complications	1 point
Occupational requirement (e.g. driver/shift worker)	1 point
**Potential total**:	**10 points**

^1^HbA1c target is < 53mmol/mol (the above target those who are less healthy)

^2^Rurality defined using participants home address and the Geographical Classification of Health [35]

^3^Younger age – due to longer potential life with diabetes and risk of complication over time

Based on assessment of the prioritisation score, and study capacity, individuals will be invited to enrol in the Hub via email or phone.

#### Baseline

After completing consent, participants will then be directed to complete a range of online questionnaires (or on paper if unable to be completed online) including the diabetes treatment satisfaction questionnaire (DTSQ), EQ-5D, and the carbohydrate counting assessment tool.

If a participant has not had a routine biochemical review completed as part of their usual diabetes care in the past 12 months, a referral will be sent to the relevant laboratories where the participant is located for collection of samples to assess: urinary albumin: creatinine ratio, lipid and/or renal studies. A request for an updated HbA1c will be sent for all participants (either laboratory or point of care). Anthropometric data (weight, height and blood pressure) included in the referral will be used as baseline data.

#### Telehealth consultation

Participants and their whānau (families) will be invited to attend a telehealth consultation with a staff member of the Hub. During the consultation, individuals will have the opportunity to discuss current commercial AID options in NZ, which at the time of this protocol development includes the Control-IQ system with the Tandem t: slim X2 insulin pump (Tandem Diabetes Care Incorporated, California, USA) and the CamAPS system (CamDiab Limited, Cambridge, England) with the mylife Ypsopump (Ypsomed Holding AG, Burgdorf, Switzerland). Other systems are commercially available in NZ but funded access is restricted so beyond the scope of this model of care. Following initial consultation, participants will select their preferred AID system including the compatible CGM. Two weeks of CGM data prior to AID commencement will be used as baseline glycaemic data. Participants will be provided with a pump start guide that includes information about starting AID, and will be used by clinical staff during education and participant follow up.

#### Preparation for commencement of AID

After selection of their preferred AID system, participants will be provided with a prescription for all equipment required prior to the pump-start training. Special Authority and prescriptions will be completed by clinical staff who have a prescribing authorisation. Prior to commencement of AID, the Hub clinical staff will determine the initial pump settings based on at least two weeks of CGM data, their recent insulin dose history, as well as physical and biochemical measurements collected at baseline. Pump start settings will be sent to the Pump Trainer at least one week prior to training.

#### Nutrition education

All individuals enrolled in the Hub will be required to complete a carbohydrate counting assessment tool online, prior to commencement of AID. For children 11 years or younger, parents will complete the assessment. Individuals with lower scores from the assessment, or those that for any reason have been identified to require dietetic follow up from the initial consultations, will be invited to attend either a structured group carbohydrate awareness education session or a one-on-one session led by a NZ Registered Dietitian. These education sessions will be optional and will occur independently to the timeline for AID start (e.g., participants may start AID prior to optional education session). Participants who attend the group sessions will be asked to complete a three question pre- and post- session self-efficacy questionnaire (each question scored on a five-point scale: How confident are you in your ability to accurately count the amount of carbohydrate in each of your meals? How important is carbohydrate counting, to you, for managing your diabetes? How confident are you in your ability to identify foods that contain carbohydrate?). Participants whose score indicates they should use fixed carbohydrate doses while awaiting Dietitian input, will be asked to complete a 3-day diet record using Easy Diet Diary to allow for calculation of fixed doses as per established and published methods [23, 36].

#### Commencement of AID (Day 0)

Participants and their whānau (families) will be invited to attend an in-person training session for commencement of AID. The training will be provided by a certified pump trainer from the pump supplier at a suitable location. To optimise resources, group training sessions will be held, however, individual training sessions will be provided where needed. Whilst in-person training sessions are encouraged, online AID training sessions will be arranged if required. Directly following the training session, participants will be contacted by a clinical staff member to provide support. Participants usual health care providers will be notified that the person is starting on AID.

#### Follow up

Once AID has commenced participants will have daily remote data review for the first two days (Day 1 and 2), one review in weeks two, three and four and one review during both the second and third months, ending at the end of the 12-week period. Follow up will be increased/decreased if clinically required and 24/7 technical support will be provided by the relevant diabetes technology company as per standard care. The content of the follow up sessions will be guided by each participants experiences and will focus on key topics of additional training used in standard care including, but not limited to: exercise, bolus adjustment, managing sick days, assessing and adjusting system settings, and optimising use of the AID system.

#### 12-weeks (primary end-point)

At 12 weeks, participants will be asked to complete the DTSQ change and EQ-5D questionnaires, and to attend their nearest laboratory for a final measurement of HbA1c. CGM data will be downloaded from participants’ online profile. Access to participant profiles will be retained on the study accounts to allow for remote review at 6-months for the purpose of data retrieval. Participants will be referred back to their usual care team at 12-weeks post AID start or earlier if stable (e.g., If TIR is > 70% or individualised TIR target is met with no significant hypoglycaemia or other concerns, the patient will be discharged before 12 weeks). This will involve a transfer of all CGM and HbA1c data back to their usual care teams to ensure optimal ongoing support. If for any reasons the Hub team do not believe discharge at 12 weeks is safe, then this will occur at a later date when appropriate.

#### Qualitative interviews

As this study will assess a new model of diabetes care in NZ, a series of qualitative studies will be completed to explore the lived experiences of participants with a particular focus on Hub satisfaction and acceptability, as well as general experiences using AID. Semi-structured interviews of 50–60 min will be conducted with a wide sample of participants enrolled in the Hub. Participants will be invited to include whānau (family) in these interviews according to their own preferences. Interviews will be conducted via video or phone call, or in-person if requested, after AID has been started and participants have been transitioned back to their usual care teams. We will conduct three qualitative sub-studies, with participants and their whānau (families), (Māori participants, Pacific participants and non-Māori, non-Pacific participants, each with a sample size of 10–15 participants). Potential participants from the main study will have the option to consent to the qualitative sub-study when completing the online consent form.

A further objective of this study is to determine whether the experience diabetes care providers obtain by being employed by the Hub (i.e., clinical staff) is effective in improving their self-efficacy in preparing, training and managing patients using AID. All clinical staff employed by the Hub will be invited to consent to participate in a qualitative interview and to complete a self-efficacy questionnaire via an online participant information and consent form. If consented, they will complete the self-efficacy questionnaire at baseline and again upon leaving the Hub, and to participate in a semi-structured interview. Demographic information will also be collected (e.g., age, sex, occupation).

#### 6-months

At 6-months, CGM data uploaded to study online accounts will be reviewed to determine if any improvements in glycaemia are maintained followed transfer back to usual care teams. After this review has been completed, participant profiles will be removed from study accounts.

### Outcomes

The primary outcome of this study is to assess the implementation effectiveness of the Hub. Secondary outcomes of this study include intervention effectiveness, safety and acceptability, psychosocial measures, nutrition education effectiveness, cost-effectiveness and clinical staff self-efficacy and intervention acceptability.

#### Implementation effectiveness

Implementation effectiveness of the Hub, at facilitating AID starts and addressing inequities in care, will be assessed by determining the number and characteristics of people who are referred and enrolled by the Hub, and the time to discharge on AID. Counts of AID starts will be compared by provider (Hub versus all others) and over time (weekly from 12 months prior to Hub implementation until study end). Incidence proportions of new AID starts, and prevalence for overall AID usage, will be estimated nationally and by region for priority populations; Māori, Pacific, those living in rural areas, and those living in areas of highest socioeconomic deprivation as measured by the NZ deprivation index [37].

#### Intervention effectiveness

Intervention effectiveness of AID will be determined by estimating participants’ changes in standard glycaemic metrics between baseline and 12-weeks including: HbA1c, time in range ([TIR], 3.9 to 10.0 mmol/L [70 to 180 mg/dL]), time in tight range ([TING/TITR] 3.9 to 7.8 mmol/L [70 to 140 mg/dL]), time below range ([TBR] < 3.9mmol/L [< 70 mg/dL]), and time < 3mmol/L (< 54 mg/dL), > 10mmol/L (> 180 mg/dL) and > 13.9mmol/L (> 250 mg/dL). CGM metrics will also be assessed between baseline and 12-weeks, and again at 24-weeks (6 months) to determine whether any benefits are maintained following discharge, These include glycaemic management index (GMI), glycaemic outcomes differentiated as 24 h, day (0600–2359 h) and night (0000–0559 h), co-efficient of variation (CV_glucose_), and mean sensor glucose +/-SD.

#### Intervention safety

Intervention safety will be assessed by the occurrence of serious adverse events (SAEs) including death and hospital admissions for diabetic ketoacidosis (DKA), severe hypoglycaemia (defined as coma or convulsion requiring assistance from others) and hyperglycaemia. Participants will be instructed to notify study staff immediately in the event of any SAE related to the study, as outlined above. Incidence will be calculated for study participants and compared to controls utilising national data captured by Health New Zealand.

#### Diabetes treatment satisfaction

The DTSQs and the DTSQ-change (DTSQc) are validated self-report measures of a participant’s current treatment satisfaction, and consist of an 8-item adult version (18 + years of age) and 12-item teen version (13–17 years of age), and a 14-item parent version that measures a parent’s satisfaction with their teen’s (ages 13–15 years) diabetes treatment [38]. The DTSQs Parent/Teen are used at baseline and follow-up to provide a ‘difference’ score for comparison. Scores can be compared between teens and their parents’ reports. The DTSQc has been developed to overcome potential ceiling effects, where respondents score near-maximum satisfaction at baseline and would therefore show little or no improvement at follow-ups [38]. Participants will complete the DTSQs at baseline and both the DTSQs and DTSQc at 12-weeks, to assess changes baseline, end of study and change in diabetes treatment satisfaction.

#### Health status

The EQ-5D-5 L (adults) and EQ-5D-Y-L (youth, ages 4–16 years of age) questionnaires provide standardised measures of health status, to describe health across a wide range of diseases [39]. The instrument comprises five dimensions (mobility, self-care, usual activities, pain/discomfort and anxiety/depression), and within each dimension there are five response levels (no problems through to extreme problems). Participants will be asked to indicate their health state by checking the box next to the appropriate response level for each dimension. The adult questionnaire has been validated in NZ and is available in English and Te Reo Māori, both options will be available depending on participant preference [40]. For the youth questionnaire, the validated version is available in English (UK) only. Participants will complete the questionnaire at baseline, and again at 12-weeks to assess the change in health status after starting on AID.

#### Nutrition education effectiveness

To assess the effect of nutrition education, of those who were invited to attend an education session (i.e., those participants with lower baseline carbohydrate counting skills), glycaemic metrics at 12-weeks will be compared for those who did, and did not attend. Participants will also be asked about their experience engaging with the study Dietitian during the qualitative interview which will provide lived experienced and contextual feedback specifically about the nutrition education provided.

#### Qualitative interviews

In brief, audio recordings from the semi-structured interviews with both participants and clinical staff will be transcribed verbatim, and recordings will be deleted thereafter. Nvivo software will be used to conduct a thematic analysis to identify Hub satisfaction and acceptability (for interviews with study participants), and factors that supported and hindered clinician self-efficacy in managing AID (for interviews with clinical staff). Themes will be compared between members of the analysis team until consensus is reached. For study participants, age, sex, insulin regimen, and duration since diagnosis will be taken from clinical records in order to describe the sample. For clinical staff, age, sex, and occupation/role will be collected in order to describe the sample. The qualitative sub-study of the experience of Māori participants in the ACCESS-AID study will be guided by the principles of decolonising methodologies [41] challenging western research practices to centre the needs, voices, and expectations of Māori participants and whānau. Interviews with Pacific participants will be managed in culturally appropriate ways with consideration to relevant Pacific health models.

#### Self efficacy questionnaire

Self-efficacy refers to an individual’s belief in their capacity to execute behaviours necessary to produce specific performance attainments [42]. In this context we would like to assess clinical staff’s self-efficacy related to managing patients with T1D who are using AID. A questionnaire has been developed specifically for this study and independently reviewed. This will be used to assess aspects of self-efficacy specifically related to AID management. This will be completed prior to the first day of employment with the Hub, and during staff members final week employed by the Hub, to assess changes in self-efficacy.

#### Existing workforce capacity building

A weekly or fortnightly drop-in webinar series is proposed to support healthcare professionals across Aotearoa in building confidence and capability in insulin pump and AID systems. Open to all clinicians with an interest in CGM and AID specifically those who have referred patients to the Hub, the series will explore a wide range of topics related to AID over time. Each one-hour session will include approximately 20 min of focused teaching on a specific aspect of pump therapy (e.g. interpreting pump data, managing exercise, conducting effective initiation appointments), followed by case-based discussion and Q&A. Case studies may be submitted in advance or brought to the session for collaborative discussion and clinical problem-solving, drawing on the expertise of the Hub’s clinical staff facilitators and peers. If no external cases are submitted, clinical staff will present relevant examples to support learning. A PowerPoint template will be provided to ensure consistent, anonymised case presentation, whether delivered by the contributing clinician or presented on their behalf. Educational content will be aligned with the forthcoming update to the Diabetes Nursing Knowledge and Skills Framework (and any other frameworks as suggested).

#### Cost-effectiveness analysis

To estimate the cost-effectiveness of the Hub, we will begin by establishing a control group comprised of individuals who initiate AID in NZ through their routine care providers. This control group will be carefully selected to match the intervention group (participants receiving care through the Hub). We will complete a cost-effectiveness analysis (cost per quality-adjusted life-years) in the intervention group compared to the control group. Markov model will be used for the cost-effectiveness analysis. The model construction and data analysis will be performed using TreeAge Pro. This study will be from the perspective of the health service in NZ, and only direct medical costs will be considered. The simulation process within the model will commence at the age of 18 years and will extend to age 100, providing a comprehensive view of the long-term impact of the Hub versus standard care.

#### Safety

SAEs will be managed as outlined in Sect. 2.6.3. All SAEs will be reported to the principal investigators of the study within one day of occurrence. Participants will also be advised to notify study staff of any adverse events related to the devices. This information will be used for internal safety monitoring. Technical support will be available to participants 24 h a day, and will be provided by device specific technical support services, as per usual care. Any device deficiencies (e.g., inaccurate labelling, device malfunction), will be recorded and reported to the device manufacturers as per usual practice.

### Statistical analysis

Pharmac, NZs pharmaceutical funding agency, estimates there are 18,000 to 21,000 people with pancreatic insufficiency (mostly T1D) living in NZ who would be eligible for funded CGM [11], of whom 85–90% were not using AID in 2023. We believe the Hub will facilitate approximately 500 new AID starts per year (provided by 2.4 full time equivalent clinical staff, per year).

A sample size of 500 or more participants will provide > 90% power, with a two-sided alpha of 0.05, to detect small changes in continuous outcomes (effect sizes ≥ 0.15) and estimate binary outcomes to within +/- 5%.

Numbers of people referred to the Hub, enrolled, and started on AID will be tabulated by the key demographic and clinical variables required for calculation of the prioritisation score as listed under Sect. 2.5.1.

Incidence proportions, stratified by region and priority subpopulations, will be calculated as the number of AID starts divided by the number of eligible people not on AID as estimated from national administrative datasets. For comparisons with other providers, an individual will be considered to have started on AID on the date they are first dispensed a subsidised pump as recorded by Health New Zealand.

Interrupted time series analysis will be used to investigate the impact that the Hub has on the rate of new AID starts nationally, and by region, Absolute numbers and incidence proportions of new AID starts will be calculated for consecutive time periods and displayed graphically. Generalised linear (Poisson) regression models will be used to estimate changes in the in the numbers and incidence of new AID starts before versus after deployment of the Hub. Differences between regions, such as workforce capacity, may be explored in models to identify facilitators and barriers to AID uptake.

Intervention effectiveness will be confirmed by descriptively summarising changes in Hub participants’ HbA1c and CGM metrics before versus after starting on AID.

National laboratory and CGM data is not available for those not enrolled in the study.

Incidence rates of adverse events will be calculated for study participants after AID start and compared to control populations utilising generalised linear regression models, adjusting for demographic factors (age, gender, ethnicity, NZ deprivation index). Controls will included study participants prior to AID start and non-study people with type one diabetes who have and have not started on AID.

## Discussion

Despite evidence supporting AID as the gold standard in managing T1D, structural and systemic barriers continue to limit access [29, 32], especially for Indigenous peoples [5, 6, 43], and those living in rural areas [31]. This study leverages our research groups specialist knowledge in advanced diabetes technology to propose a solution to an identified problem in the current diabetes health care provision. By decentralising training and support, this novel remote Hub aims to overcome workforce capacity limitations and methodological and geographical barriers. This is the first study to work in partnership with the National health care provider to implement an new model of care specific to AID. Overall this study aims to impact individuals and health care systems so that people living with diabetes, and diabetes care providers accross AoNZ experience signficant benefits from this new model of care.

Ultimately, the primary beneficiaries of this study will be people with diabetes, who should receive expedited access to the anticipated health improvements facilitated by AID, as well as reduced diabetes-related burden of care through automation of up to 50–75% of insulin dosing [1, 44]. Staff in diabetes care teams will also experience gains in training and support, which we hope enables clinical practice dissemination in the existing healthcare system. Furthermore, we anticipate that this new model of care will be cost-effective and help to reduce the long term economic burden to the NZ health system.

A key strength of this study is the focus on equitable access by prioritising people who have an elevated HbA1c, are of Māori or Pacific ethnicity, live rurally, have an occupational requirement, are younger than 25 years old, have diabetes-related complications and/or have history of severe hypoglycaemia, using the prioritisation score developed the study team. Furthermore this study provides nutrition support to participants who require additional education in this area, but without delaying AID start.

This study addresses an urgent and growing health need in NZ, and provides a framework for the roll out of a sustainable and equitable model of care that complements existing services. We view this as the future of mainstream diabetes technology training and support, especially during this period of peak uptake. Establishing a precedent and evidence for this sort of nationally delivered health care model is vital for NZ, and this work could be adapted for management of other chronic conditions.

## Data Availability

No datasets were generated or analysed during the current study.

## Declarations

### Trial registration

This study has been registered with the Australian and New Zealand Clinical Trial Regsitry (ACTRN12625000440426; 12 May 2025) and the World Health Orgnization International Clinical Trial Registry Platform (Universal Trual Number U1111-1319-6229).
